# Supplementary material for: A Critical View of the Application of the APEX Software (Aqueous Photochemistry of Environmentally-Occurring Xenobiotics) to Predict Photoreaction Kinetics in Surface Freshwaters
Source: Molecules. 2019 Dec 18;25(1):9. doi: 10.3390/molecules25010009 (PMC7017383; doi:10.3390/molecules25010009)
Supplement: Supplementary file 1 [file molecules-25-00009-s001.zip › 3.2.4_gcc-4.4.0.zip!/3.2.4_gcc-4.4.0/3.2.4_gcc-4.4.0/MSYS/doc/msys/MSYS-1.0.11-changes.rtf]

Minimal SYStem
Version 1.0.11
http://www.mingw.org/wiki/msys/

Production Release

The following changes were crafted for this release:
•	Code reduction.
•	Remove unused code.
•	More symlink reduction.
•	Modifications to msys.bat to allow -norxvt switch and to allow execution from any directory.
•	start /msys.bat
•	start /msys.bat –norxvt
•	Modifications to allow 64bit Windows to use 32bit emulation.
•	Command.com intermediate window starts in minimized mode to prevent flashy windows.
•	Internal dll identity is now msys-1.0.
•	The limitation for use of /bin and /usr/bin has been removed.
•	The chmod() function now partially works.
•	https://sourceforge.net/tracker/?func=detail&atid=102435&aid=900281&group_id=2435
•	Work around the rxvt resize freeze issue.
•	http://sourceforge.net/tracker/?func=detail&aid=921437&group_id=2435&atid=102435
•	Allow -Wl,Map,/foo/bar/baz.map to resolve to Win32 path.
•	http://sourceforge.net/tracker/?func=detail&aid=935348&group_id=2435&atid=102435
•	which script enhanced
•	Provide –a and –all
•	Provide `missing argument' and `usage' summary
•	Preserve `.exe' extensions if specified on command line
•	Provide `unknown command' diagnostic
•	Set explicit exit status code.
•	Permission Denied errors trapped
•	retry operation after x time of usleep
•	loops x number before giving up
•	helps prevent bogus errors due to clashing resource use
•	Work-around some Vista issues:
•	Continuously spawn multiple copies of the rxvt terminal, on Vista 64.
http://sourceforge.net/tracker/?func=detail&aid=1674783&group_id=2435&atid=102435
•	install.exe, install-info.exe, patch.exe fail on Vista.
http://sourceforge.net/tracker/?func=detail&aid=1711379&group_id=2435&atid=102435 
•	GNU Make now accepts both case insensitive and sensitive rules.
https://sourceforge.net/tracker/?func=detail&aid=1928876&group_id=2435&atid=102435
•	Smarter mount/umount commands.
	https://sourceforge.net/tracker/?func=detail&aid=1818517&group_id=2435&atid=352435	
•	New tools: lzma, file, paste, join, d2u, u2d.
•	The vi editor was moved to the MSYS DTK.
•	MSYS shortcut opens the Windows Console by default, to avoid the issues with pseudo-tty emulation in the rxvt terminal.


Future plans:
•	1.0.12:
•	Add options for various path methods, NOPOSIX and FASTPATH.
•	1.0.x
•	Update newlib version for 64bit file system support.
•	Modify MSYS build process to use current GCC and binutils versions.
•	x.x.x:
•	Remove unused code.
•	Restructure environment string processing so that two versions Win32 vs POSIX are kept synchronized and sorted at time of creation.
•	Rework the Heap allocation methods so that we can controll split heaps.
•	Rework the stat methods for abscense of symlink to speed up the processes.
•	Use gcc-3.x.y for build process.
•	Add classes and methods and rework existing classes and methods for improved handling of path strings.
•	Add hashing arrays to hold the pathing data cached for a period of time and allow the pathing and stat functions to use these to speed up processing.
